# The Role of Humanization and Robustness of Large Language Models in Conversational Artificial Intelligence for Individuals With Depression: A Critical Analysis

**DOI:** 10.2196/56569

**Published:** 2024-07-02

**Authors:** Andrea Ferrario, Jana Sedlakova, Manuel Trachsel

**Affiliations:** 1 Institute Biomedical Ethics and History of Medicine, University of Zurich, Zurich, Switzerland; 2 Mobiliar Lab for Analytics at ETH, ETH Zurich, Zurich, Switzerland; 3 Digital Society Initiative, University of Zurich, Zurich, Switzerland; 4 Institute for Implementation Science in Health Care, University of Zurich, Zurich, Switzerland; 5 University of Basel, Basel, Switzerland; 6 University Hospital Basel, Basel, Switzerland; 7 University Psychiatric Clinics Basel, Basel, Switzerland

**Keywords:** generative AI, large language models, large language model, LLM, LLMs, machine learning, ML, natural language processing, NLP, deep learning, depression, mental health, mental illness, mental disease, mental diseases, mental illnesses, artificial intelligence, AI, digital health, digital technology, digital intervention, digital interventions, ethics

## Abstract

Large language model (LLM)–powered services are gaining popularity in various applications due to their exceptional performance in many tasks, such as sentiment analysis and answering questions. Recently, research has been exploring their potential use in digital health contexts, particularly in the mental health domain. However, implementing LLM-enhanced conversational artificial intelligence (CAI) presents significant ethical, technical, and clinical challenges. In this viewpoint paper, we discuss 2 challenges that affect the use of LLM-enhanced CAI for individuals with mental health issues, focusing on the use case of patients with depression: the tendency to humanize LLM-enhanced CAI and their lack of contextualized robustness. Our approach is interdisciplinary, relying on considerations from philosophy, psychology, and computer science. We argue that the humanization of LLM-enhanced CAI hinges on the reflection of what it means to simulate “human-like” features with LLMs and what role these systems should play in interactions with humans. Further, ensuring the contextualization of the robustness of LLMs requires considering the specificities of language production in individuals with depression, as well as its evolution over time. Finally, we provide a series of recommendations to foster the responsible design and deployment of LLM-enhanced CAI for the therapeutic support of individuals with depression.

## Introduction

### What Are Large Language Models?

Large language models (LLMs) are a type of generative artificial intelligence (AI) that displays unprecedented performance in different downstream tasks, such as question answering and, in general, context-aware text generation [1-4]. They produce language using deep neural networks. These models consist of billions of parameters and are trained on huge amounts of data at the expense of notable computational power. LLMs have been recently popularized by services—such as OpenAI’s ChatGPT-4, Google’s BARD (now called “Gemini”), and Meta’s Llama—that are currently used by millions of people every day, experts, and laypeople alike. These services are essentially conversational AI (CAI) enhanced with LLMs. They offer a more human-like, natural, and context-relevant interaction than other technological applications such as rule-based conversational agents (ie, traditional “chatbots”). They hold the potential to transform how we engage in conversations and manage the information therein. Consequently, they are expected to become much more widely adopted in different professional fields, research, and society alike.

### LLMs in Health Care and Mental Health Applications

In health care, applications of LLMs are manifold, spanning from clinical research and processes to physician-patient relations [5-10]. For instance, LLMs can improve clinical processes by automating the generation of administrative text [1,5]. Physician-patient relations could benefit from the use of LLM-enhanced patient decision aids and interventions that could support therapy and improve shared decision-making [1]. Context-relevant and personalized conversations with an LLM-enhanced CAI show the potential to promote patients’ empowerment and individuals’ reflection around their personal values and preferences for different health care scenarios in a way that is not possible with current methods, for example, filling out legal documents such as advance directives [11-13].

In the mental health domain, the use of CAI is no novelty. The very first chatbot ELIZA, was developed in 1966, and played the role of a digital psychotherapist [14]. Six decades later, it is possible to develop and test LLM-enhanced CAI leveraging an ample body of knowledge and use cases. In the mental health domain, CAIs are currently used as patient therapeutic support, for example, a simple psychotherapy, such as cognitive behavioral exercises [15]. Given their noteworthy ability to process and produce language, the use of LLMs holds the potential to provide more context-aware and effective psychotherapeutic support to their users than traditional CAI. In fact, once embedded in CAI, designers can instruct LLMs to provide a nonjudgmental, readily available platform for vulnerable individuals to discuss their feelings and mental health struggles as well as practice skills that they learned in a therapeutic session.

CAI is also used to collect data of patients with mental health disorders, carry out initial triage processes, and provide treatment recommendations [6]. Here, LLM-enhanced CAI could process written or spoken responses of patients with mental health disorders to support therapists in their diagnostics or track mental health changes in patients over time. They could also generate personalized treatment recommendations by taking an individual’s mental health history, their symptoms, values, and care preferences as input. By collecting data on the web, such as social media posts or chat logs, LLMs could help detect signs of emotional distress and detect mental health issues promptly. To this end, preliminary results show that ChatGPT-3.5 achieves good performance at detecting stress and depression in written statements on web-based forums [16]. These results suggest that ChatGPT-3.5–enhanced CAI could be in the future used in psychotherapeutic scenarios, for example, among patients with depression [16].

### Ethical, Technical, and Clinical Risks Posed by LLMs

Emerging technology, including LLMs, are not immune to risks. They stem from conceptual, ethical, technical, and clinical considerations and become especially important in domains such as mental health care. They comprise ethical issues linked to bias, global digital divide, trustworthiness, black-box nature and validation, and the generalizability challenges in training and deploying LLMs [1,4,17].

In addition, authors are addressing different ethical problems associated with the use of LLMs in mental health applications. For instance, Cabrera et al [18] relate these challenges to the 4 principles of biomedical ethics with emphasis on data privacy, confidentiality, avoiding manipulation, and safety. Similarly, Yang et al [19] emphasize the necessity of carefully evaluating LLM-enhanced CAI in mental health applications by advocating the use of explainability methods to make the outcomes of LLMs more transparent. They also suggest complementing the use of LLMs with additional sources of information, such as emotional cues and cause-effect reasoning to enhance the quality of mental health support [19]. Further, Thirunavukarasu et al [1] emphasized the importance of using domain-specific data to fine-tune LLMs to validate LLM-enhanced applications with real clinical use cases. Finally, research is also investigating these challenges, with a focus on the perspective of end users of these systems, for example, individuals engaged in digitally assisted therapies. To this end, Weidinger et al [4] identify 6 areas of risk and potential harm to users of LLMs, including issues such as discrimination, privacy risks, misinformation, and human-computer interaction challenges [4]. In particular, they emphasize the risks for the users of LLM-enhanced services, which stem from their “human-like” design.

### Mental Health Use Case: LLM-Enhanced CAI to Support Individuals With Depression

In summary, regarding the use of LLMs, research needs to address a mix of familiar and novel conceptual, ethical, technical, and clinical issues. To improve our understanding of these challenges, we need to examine LLMs within specific domains. This approach becomes particularly pertinent in the mental health domain, where the high sensitivity of the use cases underscores the imperative for a responsible and effective implementation of LLMs in CAI systems that provide therapeutic support to vulnerable individuals.

In this work, we focus on the scenario where LLM-enhanced CAI systems are used in the mental health domain to promote therapeutic support focusing on individuals with depression. The rationale behind selecting this use case is as follows. First, depression affects over 300 million people and the World Health Organization identifies it as the largest single contributor to global disability [20]. From an economic standpoint, for instance, studies have estimated the economic impact of depression to be 1.6% of the US gross domestic product [21]. Further, burnout is a major issue among psychiatrists [22]. Then, it is imperative to integrate technology-mediated interventions alongside traditional therapy methods to enhance accessibility and effectiveness of mental health services. Here, the use of CAI for patients with depression is widespread and supported by an ample body of scientific evidence [15]. More recently, research has also started exploring the use of LLMs for addressing depression [3,23].

This said, it is still an open avenue of research to delineate the perimeter for the responsible use of LLMs in scenarios involving individuals with depression. Therefore, in this work, we contribute to research on the responsible design, integration, and use of generative AI in mental health by focusing on 2 challenges that affect all scenarios where individuals with depression interact with LLM-enhanced CAI. In particular, we address challenges that pertain the (1) humanization of LLM-enhanced CAI (philosophy and psychology) and (2) contextualization of the robustness desideratum (computer science).

Our approach is interdisciplinary and relies on theories and methods from philosophy, ethics, psychology, and computer science. Our aim is to conceptually analyze 2 topics that are underexplored in the literature on LLMs and their applications in mental health care despite their importance while highlighting their risks. With our analysis, we provide a critical perspective on the CAI-specific trend of humanizing CAI and the problem of treating the robustness of LLM-based CAI systems as a context-independent challenge. We cross the boundaries of the disciplines to show how our conceptual analysis can be informative for issues in computer science and health research [24]. In fact, when properly translated, our conceptual analysis can generate valuable insights in empirical disciplines [25]. Finally, we discuss recommendations to promote the responsible use of LLM-enhanced CAI in the mental health domain.

## Use of LLM-Enhanced CAIs by Individuals With Depression: 2 Challenges

### Humanizing LLM-Enhanced CAI: a Philosophical and Psychological Perspective

Humanization is intended to develop CAI with the goal of simulating human abilities and traits, such as humor, empathy, and politeness. It is different from anthropomorphism, which refers to users’ tendency to attribute CAI with human abilities and traits [26,27], although the CAI does need to be intentionally designed to mimic humans [26]. In practice, humanization of CAI is achieved by developing verbal, nonverbal, visual, and relational cues to make the system more human-like [28,29]. For example, CAI can have a persona (relational cues) that simulates certain human personalities, such as being a friend or therapist. This persona is often implemented in the avatar (visual cues), the informal language (linguistic cues), and emojis (nonverbal cues) used by the CAI. Empirical studies suggest that the humanized abilities and characteristics of CAI, such as reciprocity or giving empathetic responses, has positive outcomes on digital health interactions, such as improved user experience and the formation of relationships with CAI, trust, or better engagement [27,30-33]. These outcomes are particularly relevant in mental health care applications, where therapeutic relationships with the CAI promote therapeutic effectiveness, and high levels of user engagement might limit drop-outs [34,35]. However, research studies lack consistency in conceptualizing humanization and manipulating different cues [26]. In fact, there is no systematic inquiry to understand the extent to which specific cues lead to specific outcomes. More investigations are needed to understand the underlying mechanisms of measured effects (eg, linguistic vs nonlinguistic cues) and assess how these differ on the basis of design choices of humanized CAI.

LLMs can simulate context-aware conversations with their users and demonstrate an ability to adopt conversational personas. This allows LLM-enhanced CAI displaying features that strongly resemble human abilities and characteristics to an unprecedented level [36]. As noted by Shanahan et al [36], LLMs are fundamentally dialogue agents that role-play an ample variety of human-like characters [36]. While there is a generally positive view of humanizing CAI in various domains, we argue for a critical view of humanization efforts. Particularly, the effort of humanizing LLM-enhanced CAI in mental health applications presents serious challenges that must be tackled. Research highlights concerns about the safety of vulnerable users interacting with “human-like” systems [4]. This perspective on humanization emphasizes the potential risks and challenges stemming from the interactions between LLM-enhanced CAI and users, such as individuals with depression. However, there appears to be a lack of theoretical perspectives and clarification on humanization although humanizing concepts are fundamentally rooted in describing and developing AI systems [27,37]. This theoretical clarification could inform the responsible development of these systems, particularly in mental health applications. In what follows, we address this gap by relying on philosophical, ethical, and psychological considerations.

#### Conceptual Considerations

First, a conceptual problem underlies the development of LLM-enhanced and “humanized” CAI. We argue that it is important to maintain a distinction between the characteristics and traits simulated by these systems and the human qualities that are referred to using the same concepts and terminology. Simulated abilities and characteristics of LLM-based CAI are not the same as the original human abilities and characteristics. There are fundamental differences between humans and AI that further problematize an uncritical adoption of human concepts in the context of CAI. These problems have been addressed in different research domains. For instance, Bender et al [38] focus on the difference between synthetic language produced by LLM and human natural language by arguing that LLMs are “stochastic parrots” producing language, but not understanding it. Felin and Holweg [39] similarly argue by reporting differences in human cognition and computation processes of AI. Such arguments are often based on linguistic, philosophical, and psychological knowledge about human cognition, understanding and belief systems that are based on meaning, intentions, theory-based logic, and experience and are embedded in social and normative space [39-43]. In philosophy, the argumentation can stem from the analysis of such concepts as rational and moral agency that are not present in CAI, but are inherent in humans and their activities such as conversations [43]. Another strategy could be to analyze CAI as a different system from humans and by showing the limits of their models that cannot reach the complexity of human intelligence as reported by Landgrebe and Smith [44]. All these considerations have in common the fact that they provide a diversity of arguments for the position that CAI’s simulated abilities and characteristics differ from humans [45,46]. In line with this literature, we argue for careful descriptions of CAI when human concepts are used. Such human concepts and terms such as being genuinely “empathetic,” “compassionate,” “inclusive,” “polite,” or “authoritative” mean something different when applied to CAI. If possible, CAI should be described more appropriately to avoid misconceptions and conceptual confusion. In the next subsection, we will outline problems and risks that might stem from such misconceptions and conceptual confusion.

In mental health literature, we found specific examples criticizing the adoption of human concepts for LLM-enhanced CAI. A good and common example is “empathy,” which is a key component of psychotherapy [47,48]. Recently, researchers investigated the simulation of an LLM-based “empathetic therapist” with individuals with depression [16]. The fact that an LLM-enhanced CAI can generate a seemingly empathetic response is substantially different from a human actually expressing empathy [49]. This ability is linked with someone’s personality and emotional profile, shared social space, and lived experiences [47]. To be empathetic means to achieve genuine *understanding* of what another person is experiencing or attempting to express. Empathy includes active listening, asking targeted questions, and expressing genuine concern effectively addressing emotional needs [47]. These activities lie beyond the capabilities of LLMs, which are disembodied statistical processes. Most importantly, LLMs do not understand users’ inputs and, in particular, do not understand their semantics [50,51], despite representing a vast body of information in a neural network.

Here, understanding (eg, a statement) is a crucial epistemic accomplishment arising from a myriad of complex cognitive activities that result in grasping meaning (eg, of statements and their components) and causal relationships, testing alternative knowledge pathways, on top of providing well-grounded reasons for each of those. Furthermore, understanding emerges as the culmination of intricate processes that are socially and normatively embedded [52]. This attainment is fostered by virtues, such as perseverance, precision, and epistemic humility among others. These characterize, in particular, how human experts in a research domain structure knowledge and seek understanding. In contrast, LLMs compute answers through statistical processes that simply do not take into account the meaning of user’s prompts [39]. As a result, understanding escapes the statistical manipulations that characterize the logic of LLMs [53,54]. In a nutshell, displaying—sometimes successful, as LLMs do hallucinate and generate “fake” references and justifications—ability to manipulate structured information does not guarantee understanding.

Vulnerable patients with depression may potentially misinterpret CAI as empathetic and caring, potentially leading to unrealistic expectations such as warmth and acceptance [55]. Due to CAI’s limitations, such misconceptions could reinforce negative beliefs and worsen emotional states. Since LLMs lack understanding of user inputs, they may respond inappropriately, misunderstanding the nuances of individual situations. This could further reinforce negative feelings or isolation in patients with depression. This point is particularly relevant for designers and therapists who need to test the capabilities of LLMs before promoting their use for digital therapy with vulnerable individuals. Differently from current research [4], we emphasize that humanization is at first a challenge for those who design and promote these systems, before becoming a risk for those who use the technology. The key here is to understand that the ability of LLMs to generate empathetic output, as opposed to being apathetic, indifferent, and insensitive in conversations, descends from the computation of empirical probabilities of “next words,” given the user prompt and their training on a massive amount of documents [36]. In fact, under the hood, LLMs perform autocomplete functions of search engines [50]. These remarks help in characterizing the limits of the humanization of LLMs and they hold true also for other characteristics and traits that LLMs attempt to simulate. This includes, in particular, the quality of being an “expert” in a domain, for example, a specialist in the treatment of depression among adolescents, and, in virtue of this, being perceived as a digital therapist, instead of a therapeutic support system [53-55].

In summary, philosophy and psychology guide us in recognizing the substantive differences between humans and humanized LLM-enhanced CAI. This helps to assess the limits of this endeavor, identify the correct roles these systems can play in interactions with humans, and, eventually mitigate misconceptions and overtrust in these systems [4]. The issue of humanization needs more in-depth analysis, including the exploration of how the human attributes assigned to LLM-enhanced CAI influence and guide patients in shaping their behavior and responses within a conversation.

#### Normative and Ethical Implications of the Conceptual Problem

The conceptual confusion of ascribing human-like abilities to LLM-enhanced CAI is linked with important normative and ethical risks, which pertain to responsibility, commitments, and rights. Overall, interpersonal conversations are social and normative activities that are embedded in a set of values, norms, and virtues [42]. This is particularly true in the case of therapeutic relationships that are guided by sets of values and norms to ensure a safe environment and therapeutic process for patients [56-59]. Such human abilities as empathy or understanding are part of this normative and professional setting. Psychiatrists and psychotherapists who do not follow professional conduct guidelines when treating individuals with depression risk causing medical emergencies for their patients—a situation that could lead to disciplinary actions against them.

In the case of humanized LLM-enhanced CAI, there is a gap between what the system appears to be, for example, being compassionate, and what normative criteria this ability should meet and cannot be met by CAI—criteria that are fulfilled by human therapists instead. Hence, when CAI simulates abilities such as empathy or understanding, these are not part of the normative setting as they are in the case of human experts. This CAI can lead to risks among individuals with depression. For instance, if an LLM’s response lacks compassion during a conversation with a user with depression, this may worsen their condition, even leading to self-harm. An LLM-enhanced CAI may not encode cultural nuances and the uniqueness of individual experiences in its outputs while its biases significantly influence how the system presents and discusses knowledge with patients. This can contribute to “epistemic injustice” [60], making individuals with depression potentially feel more isolated and their perspectives undervalued and misunderstood. In addition, human experts—for instance, psychiatrists—have epistemic duties, including being truthful and justifying their beliefs [43]. In contrast, LLMs lack these commitments [40].

Further, this “normativity gap” leads to a problem of assigning responsibility and defining how to approach failures in a conversation with patients. There is a difference between addressing the ethical consequences of technical failures of a computer system, for example, numerical errors and inaccurate predictions, and dealing with the issues that arise from a faulty implementation of humanizing features. On the one hand, technical errors in computer systems are clearly defined, objectively measured, and traced, facilitating the definition of their sanctions. On the other hand, what does it mean that the LLM-enhanced CAI was not empathetic in a given conversation? Was it not empathetic *enough*? According to which objective measures of empathy? Did the lack of empathy persist in the conversation long enough to consider applying sanctions? The humanization of LLM-enhanced CAI involves complexities that are not fully understood even in interpersonal interactions, where ambiguous, inappropriate or unprofessional questions and answers may occur, and the applicability of sanctions is unclear.

In summary, despite the current trend of humanizing LLM-enhanced CAI, it is questionable to what extent such humanization is necessary and helpful as it poses theoretical and ethical challenges. It remains an open question whether there is an ethically acceptable, safe, and beneficial degree of humanization for these systems. Philosophy and psychology can help frame the problem, which highlights a particularly important gap of the responsible design and development of AI in mental health care [61].

### Contextualizing the Robustness of LLMs Used by Individuals With Depression: a Computer Science Perspective

#### The Robustness of LLMs

Robustness refers to the ability of machine learning models to withstand “perturbations” that may affect their performance [62-64]. It is a general model capability that becomes essential for ensuring the reliability of machine learning models in real-world applications. Interestingly, robustness is a multidimensional concept that is currently lacking a one-size-fits-all definition. Rather, research discusses what a robust model *should do* [62,65-68], investigating how a model should resist different types of perturbations, such as those affecting its input data, data distributions over time, and the model structure. In fact, a robust machine learning model computes predictions that do not vary disproportionately in case of perturbed inputs. Further, it retains accuracy in the presence of distributional shift [69] and is not affected by small changes in its constitutive structure. In summary, robustness is a key requirement for trustworthy AI. It can also be extended to comprise algorithms that provide explanations of machine learning models’ predictions [65,66,68,70]. In this case, robust explanations are not altered by the perturbation of data inputs and are stable over time.

In the case of LLMs, the high-level desideratum of robustness seems to gain an extra level of complexity [63,64]. In fact, when discussing what robust LLMs should do, we need to consider the peculiar way these models compute their predictions, namely, using prompt-based queries [71]. Here, a prompt is structured information—often, a text snippet—that users offer as an instruction to the LLM and which is often accompanied by one or more examples to guide the model (“in-context learning” or “few-shot prompting” procedure) [71,72]. For example, a prompt for an LLM used in an application to investigate how patients with depression communicate with CAI may look like this: “Classify the following sentence in either normal or alerting: [s].” Here, the example [s] is the patient’s utterance: “Today, I felt more useless than usual and nobody knows it.”

Broadly speaking, an LLM is robust if its predictions display an appropriate level of sensitivity to the changes that may affect its prompts and examples. With a robust LLM, similar prompts and examples should lead to similar predictions, among others. This said, research has a long way to go before this promise can become reality. An increasing body of literature shows that commonly available LLMs, for example, T5, Vicuna, Llama 2, and ChatGPT-3.5 [73], generally display a low level of robustness. These models are highly sensitive to different types of perturbations, named “prompt-targeting adversarial attacks” [73]. These comprise switching the order of few-shot examples and semantic-preserving variations, such as adding a few typographical errors, replacing words by synonyms or back translating the prompt itself and its examples [73]. As a result, a few empirical studies show that prompt-targeting adversarial attacks can lead to substantially different LLM predictions, indicating an overall lack of robustness across a variety of downstream tasks, such as text classification and generation [73].

Finally, from an ethical perspective, the lack of robustness of LLMs is a source of different issues. Nonrobust models lead to unreliable decision-making, that is, they increase the risk of making inconsistent or erroneous decisions that can harm those affected by them. For instance, LLMs could provide misdiagnosis and share information that does not align with clinical practices, show the inability to detect and respond to nuances in language that indicate a mental health crisis (such as expressions of suicidal ideation or severe distress), and offer appropriate and timely crisis intervention resources. Finally, training on large corpora of text may lead LLMs to perpetuate forms of stigmatization against individuals affected by mental health issues (despite fine-tuning on documents from the psychiatry domain).

They may also lead to unwanted cases of bias and discrimination and pose serious concern to the privacy of individuals’ information. Nonrobust models can be tricked to reveal personal information. Finally, erratic or nonrobust model behavior affects their overall transparency levels. These ethical concerns are particularly relevant in high-stakes scenarios, such as those where LLMs are deployed to support the mental health of vulnerable individuals.

#### Contextualizing the Robustness of LLMs

Current approaches to ensuring the robustness of LLMs lack proper contextualization: they are not targeted to any specific scenario of human-LLM interaction. While it is beneficial that LLM predictions remain consistent even when prompted in similar ways or when the order of the LLM examples changed, as suggested by the emerging literature on prompt-targeting adversarial attacks [73], this alone is insufficient for the ethically responsible use of LLMs in high-risk applications, such as in scenarios involving mental health support for patients with depression. In these cases, we argue that it is necessary that LLMs’ robustness is tailored to align with the specific language characteristics—and their variations over time—of the model users, specifically, patients with depression. In other words, an appropriately robust LLM to be used by individuals with depression should detect their lexical, syntactic, cultural, and content-related language patterns, while retaining the ability of not being affected by more general adversarial attacks [73], as suggested by the high-level desideratum of robustness. In summary, the LLM should provide accurate outputs that are (1) not affected by spurious linguistic variations in the prompts and examples provided by its users and (2) tailored to the context in which the interaction takes place. This calls for the design of prompt-targeting *contextualized* adversarial attacks and the assessment of the *contextualized* robustness of LLMs, rather than the investigation of general, domain-unspecific robustness constraints.

**Figure 1.**
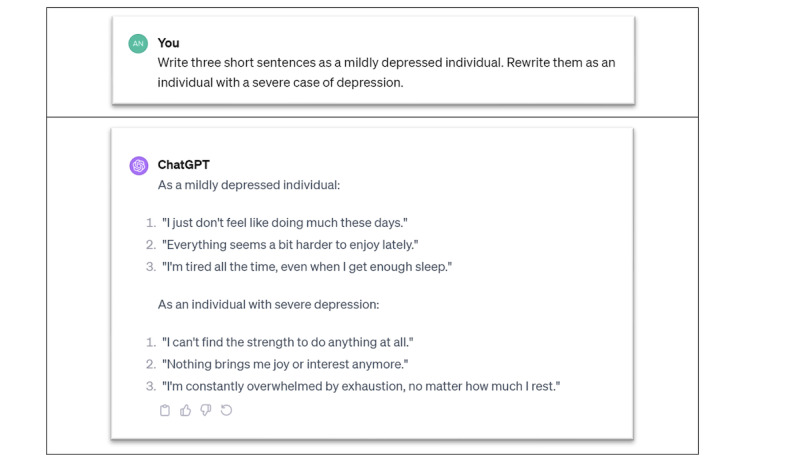
Examples of utterances by patients with mild depression and those with severe depression generated by ChatGPT-4 (prompt and answers from December 2023).

Research on depression has already identified a few linguistic patterns that may help in this regard. On average, patients with depression make more and longer pauses than healthy individuals when they communicate [74]. Further, they display a lower pitch, more monotonous speech, and slower utterance production [75,76] (notably, the importance of slowed speech is emphasized in the Patient Health Questionnaire–9 self-assessed depression report [76]). Similarly, the analysis of transcripts of utterances of individuals with depression shows that patients with depression use more modifying adverbs, first-person and personal pronouns, and more verbal utterances [76-78]. Further, individuals with depression and healthy individuals show differences in the use of past tense, causation, achievement, and death words [76], using simpler sentence structures and reduced linguistic complexity, as well as exhibiting rumination and self-focus in their language [79,80]. The instability of words associated with negative emotion predicts depression in textual production on social media as well [81]. We also note that these patterns may vary over time, as the patient may go through different stages of depression. In Figure 1, we show a few examples of such variations we generated with ChatGPT-4. In summary, robust LLMs to be used for therapy for individuals with depression should be able to correctly identify their linguistic patterns and react to their evolution appropriately. This observation is reinforced by the fact that language is a dynamic process that changes over time. New idioms, metaphors, or shifts in meaning regularly take place, and LLMs need to be aligned with the dynamics of language production. Here, the risk is to promote “frozen” narratives and linguistic patterns that do not reflect the evolution of patient narratives over time.

From a technical perspective, making LLMs contextually robust requires their fine-tuning them with high-quality, curated data. Currently, obtaining such data for patients with depression is challenging, with most available examples coming from social media platforms such as Twitter or Reddit [82]. To the best of our knowledge, there exists no publicly available data set of conversations of patients with depression from consultations with therapists, therapeutic CAI, or other agents in everyday life. Additionally, we cannot easily improve proprietary LLMs. This is a serious problem, as a recent study shows that ChatGPT-3.5 is not robust enough for conversations with individuals with symptoms of anxiety or depression, as the LLMs suggested medications to its users; medications should be taken under the guidance of a psychiatrist [83].

In summary, understanding and achieving the contextualized robustness of LLMs is crucial for the responsible use of LLM-enhanced CAI among individuals with depression. While computer science offers methodologies to formalize, evaluate, and satisfy this requirement, their effectiveness is limited by the availability of necessary resources, primarily therapy-relevant data, which are currently lacking.

### Toward Responsible Use of LLMs in Therapeutic Settings Involving Individuals With Depression

The complexities of humanization and contextualized robustness appear to temper the initial enthusiasm surrounding LLMs. The problems affecting humanization we discussed in the previous sections seem to be at odds with the very essence of LLMs, namely, to role-play different personas. Meanwhile, we noted that achieving contextualized robustness requires thorough fine-tuning and comprehensive testing. Moreover, this process must be grounded in a deep understanding of how language production and usage evolve over time among the users of these systems.

The importance of addressing the risks associated with humanization and the absence of contextual robustness is underscored by real-world incidents involving individuals with depression using LLM-enhanced CAI. There have been several instances, reported in various media, where LLM-enhanced CAI provided support for mental health issues but instead encouraged self-harm or offered detrimental advice [84,85]. A Belgian man with depression committed suicide following conversations with ChatGPT-3.5 [85]. Recently, Kumar et al [86] commented on the case of a user with depression, who, during a crisis, was able to insert a sequence of words in their prompt that bypassed the LLM’s safety-guards and generated harmful content [86]. In fact, the LLM returned detailed instructions on how to commit different types of self-harm [86]. Further, authors show that certain prompts result in ChatGPT-3.5 prescribing medications to individuals with anxiety or depression symptoms, despite medications that should be taken under the guidance of a therapist [83]. In addition, the vulnerability of LLM-enhanced CAI to attacks and content manipulation can lead to the generation of offensive, inappropriate, or objectionable responses; the provision of incorrect information; and discriminatory recommendations. These events show potential of causing either discomfort, harm, or even acute detriment to users [87].

Finally, it is argued that humanization may invite and actively nudge patients to react to its cues [4]. LLM-enhanced CAI are persuasive to their users and can perform a variety of emotional manipulations. These may lead to inappropriate reliance on these systems or overtrusting them [88], reinforcing bias, and overestimating their capabilities, including expecting unrealistic behavioral change [4,89].

Given the challenges discussed in this viewpoint paper, the path toward a responsible development and use of LLM-enhanced CAI in therapeutic settings involving individuals with depression appears to be quite challenging. Here, we agree with Cheng et al [90], who promote the idea of using LLM-enhanced CAI as an assistant to mental health professionals in providing patient care [90]. Further, they emphasize the need for routine monitoring of patients and the systems to address emerging challenges in a timely manner [90]. However, we disagree with the authors when they suggest that, from an ethical standpoint, psychiatrists should take full responsibility for any detriment to patients interacting with the LLM-enhanced CAI [90]. In fact, this claim would be justified if psychiatrists could understand these systems in depth. However, it is unlikely that psychiatrists, despite their expertise in mental health, would possess an in-depth understanding of the workings of such advanced technology.

In summary, an interdisciplinary approach to the responsible use of LLM-enhanced CAI in therapeutic settings involving users with depression is essential, encompassing both the social and technological aspects of CAI development and application [46,91]. This approach should integrate theoretical and practical perspectives from psychiatry, ethics, philosophy, computer science, and user experience design, ensuring a balanced and informed development of these technologies. These perspectives could help address the risks posed by the humanization of these systems and the lack of contextualized robustness, by suggesting ways to inform, instruct, and educate developers and users (including therapists) about the conceptual nuances of normative concepts, such as expertise, and the characteristics of language production of individuals with depression.

One practical measure to manage the risks stemming from the humanization of LLM-enhanced CAI could be incorporating disclaimers and a short conversation at the start of therapy sessions with the system. The measures would outline the capabilities and theoretical limitations of CAI, helping users in accurately setting their expectations from the interaction with the systems. Revisiting these disclaimers and conversations periodically, especially in long-term use, could reinforce users’ understanding and help them manage their expectations effectively over time.

To contextualize the robustness of LLM-enhanced CAI, researchers could collect data from different cohorts of patients with depression interacting with the system in controlled settings. They could augment these data by other sources, including survey data and clinical information to improve the accuracy of the LLMs. Further, identifying contextual features that help LLMs recognize the patients’ emotional states, triggers, or history can further improve the accuracy and contextual robustness of the models over time. These features may include over time sentiment analysis, trigger recognition, environmental information, and audio and visual cues. Therapists and patients could review these interactions to correct inaccurate suggestions and address the issues they may have caused in a timely manner. This procedure, which necessitates the active involvement of both clinical experts and patients, is undeniably time-consuming but indispensable. Moreover, it hinges on a controlled setting that may not capture all aspects of the interactions between patients with depression and LLM-enhanced CAI in everyday life. However, this is a first step to assess the risk of deploying “brittle” LLMs in clinical practice.

Finally, to responsibly use LLM-enhanced CAI with patients with depression, it is important to rigorously examine its long-term effects. Developing and adhering to strict standards for the creation and implementation of these systems is necessary, mirroring the evidence-based approach of mental health care, where interventions undergo thorough testing, including randomized controlled trials. A structured framework, akin to those used in the development and assessment of patient decision-making tools [92,93], could greatly benefit the development and application of LLM-enhanced CAI. Guidelines that address the humanization of these systems and ensure their contextual robustness should be central to this framework.

### Ethical Considerations

This study was exempt from ethical review as no human participants were involved.

## Conclusions

The use of LLMs in mental health applications presents numerous conceptual, ethical, technical, and challenges. In this work, we have outlined 2 challenges that impede the responsible use of LLMs in applications involving patients with depression: the accentuation of human-like qualities of LLM-enhanced CAI and the lack of contextualized robustness. These challenges warrant comprehensive consideration and a proactive approach to ensure the responsible and effective integration of LLMs in mental health settings. While human-like qualities may enhance user engagement, it is imperative to strike a balance when a simulation of human characteristics and abilities does not increase ethical risks and their effects are well understood. A responsible approach involves clearly communicating to users that they are interacting with AI-based tools and what this exactly means, enabling them to make informed decisions about the assistance they receive and being aware of their limitations as well as differences from human conversation.

Further, LLMs should be adept at understanding and adapting to the specific linguistic, cultural, and emotional nuances of individuals dealing with mental health issues. Robustness, in this context, involves not only maintaining coherence in responses but also sensitively addressing the unique needs of each user. Ethical guidelines should emphasize the development and validation of LLMs with a focus on contextual sensitivity. It is vital to establish a framework that delineates the roles of AI developers, health care providers, and users in ensuring the well-being of those seeking mental health support.

## Abbreviations

AI: artificial intelligence
CAI: conversational artificial intelligence
LLM: large language model
